# Circulating osteoprotegerin is associated with chronic kidney disease in hypertensive patients

**DOI:** 10.1186/s12882-017-0625-3

**Published:** 2017-07-06

**Authors:** Stella Bernardi, Barbara Toffoli, Fleur Bossi, Riccardo Candido, Elisabetta Stenner, Renzo Carretta, Fabio Barbone, Bruno Fabris

**Affiliations:** 1 Department of Medical, Surgical and Health Sciences, University of Trieste, Cattinara Teaching Hospital, Strada di Fiume, Trieste, 34100 Italy; 2 Azienda Sanitaria Universitaria Integrata di Trieste (ASUITS), Strada di Fiume, Trieste, 34100 Italy; 3 IRCCS Burlo Garofolo, Via dell’Istria, Trieste, 34100 Italy

**Keywords:** Osteoprotegerin, Hypertension, Chronic kidney disease, Biomarkers

## Abstract

**Background:**

Osteoprotegerin (OPG) is a glycoprotein that plays an important regulatory role in the skeletal, vascular, and immune system. It has been shown that OPG predicts chronic kidney disease (CKD) in diabetic patients. We hypothesized that OPG could be a risk marker of CKD development also in non-diabetic hypertensive patients.

**Methods:**

A case-control study was carried out to measure circulating OPG levels in 42 hypertensive patients with CKD and in 141 hypertensive patients without CKD. A potential relationship between OPG and the presence of CKD was investigated and a receiver-operating characteristic (ROC) curve was designed thereafter to identify a cut-off value of OPG that best explained the presence of CKD. Secondly, to evaluate whether OPG increase could affect the kidney, 18 C57BL/6J mice were randomized to be treated with saline or recombinant OPG every 3 weeks for 12 weeks.

**Results:**

Circulating OPG levels were significantly higher in hypertensive patients with CKD, and there was a significant inverse association between OPG and renal function, that was independent from other variables. ROC analysis showed that OPG levels had a high statistically predictive value on CKD in hypertensive patients, which was greater than that of hypertension. The OPG best cut-off value associated with CKD was 1109.19 ng/L. In the experimental study, OPG delivery significantly increased the gene expression of pro-inflammatory and pro-fibrotic mediators, as well as the glomerular nitrosylation of proteins.

**Conclusions:**

This study shows that OPG is associated with CKD in hypertensive patients, where it might have a higher predictive value than that of hypertension for CKD development. Secondly, we found that OPG delivery significantly increased the expression of molecular pathways involved in kidney damage. Further longitudinal studies are needed not only to evaluate whether OPG predicts CKD development but also to clarify whether OPG should be considered a risk factor for CKD.

**Electronic supplementary material:**

The online version of this article (doi:10.1186/s12882-017-0625-3) contains supplementary material, which is available to authorized users.

## Background

Osteoprotegerin (OPG) is a circulating glycoprotein that acts as a cytokine decoy receptor and antagonizes receptor activator for nuclear factor kB ligand (RANKL) and TNF-related apoptosis-inducing ligand (TRAIL) [1]. Initially, due to its ability to block RANKL and to inhibit bone reabsorption, OPG was considered as one of the key regulators of bone turnover [2]. Then, it has become increasingly clear that OPG exerted also other actions, involving the immune and the cardiovascular system [3].

Today, OPG is considered a risk marker of cardiovascular diseases (CVD) [4]. OPG levels are positively correlated with markers of vascular damage such as endothelial dysfunction [5, 6], vascular stiffness [7], and coronary calcification [8], as well as with the presence and severity of coronary artery disease [9, 10]. In addition, OPG is associated with the risk of future coronary artery disease [11, 12], heart failure [13], and with the incidence of cardiovascular [12, 14] and all-cause mortality [15, 16], not only in patients with coronary artery disease [14] but also in the general population [17].

Animal studies, which help understand the mechanisms of diseases, are conflicting on the effect of OPG on the cardiovascular system. Although initially OPG deficiency resulted in significant medial calcification of the aorta and the arteries [18, 19], our group has shown that the delivery of OPG increased atherosclerosis extension [20], suggesting that this molecule could actually promote atherosclerosis. To date, it remains unclear whether OPG increase should be considered as a risk factor rather than just a risk marker of CVD [21].

As for the kidney, it has been show that OPG is increased in patients with non-diabetic [22, 23] and diabetic chronic kidney disease (CKD) [24–26], where it predicts deterioration of kidney function, vascular events, cardiovascular and all-cause mortality [25]. Consistent with it, it has been recently reported that elevated OPG is associated with increased 5- and 10-year risk of rapid renal decline, renal disease hospitalization, and/or deaths in elderly women [27]. Overall, these studies, mostly carried out in diabetic patients, suggest that OPG could be a biomarker for CKD progression, as reviewed in [28, 29]. Nevertheless, it remains unclear whether a similar association is present also in hypertensive patients. Moreover, the direct effects of OPG on the kidney remain largely unknown.

Based on these premises, the aims of this study were to evaluate the relationship between OPG and CKD in a group of patients with essential hypertension, and to evaluate whether OPG increase could damage directly the kidney in an experimental setting.

## Materials and methods

### Clinical study

#### Study protocol

A case-control study was carried out to measure circulating OPG levels in hypertensive patients with CKD and in hypertensive patients without CKD. For this purpose, a total of 42 non-diabetic hypertensive patients with CKD (CKD) and 141 hypertensive patients with no CKD (CNT), were consecutively enrolled over two years from the subjects referring to four hospital-based Hypertension Centres [30]. CKD patients had: (a) diagnosis of moderate to severe essential hypertension (systolic blood pressure > 160 mmHg and diastolic blood pressure > 100 mmHg) prior to the development of CKD (GFR <60 mL/min/1.73 mm^2^); (b) onset of CKD before the age of 65 years; (c) low-grade proteinuria (<2 g/24 h); (d) no history of nephrotoxic exposure, congenital or intrinsic renal disease, and systemic illness associated with renal damage; (e) biopsy-proven diagnosis of nephroangiosclerosis, when available (28%). CNT patients were required to have a diagnosis of essential hypertension (systolic blood pressure > 160 mmHg and diastolic blood pressure > 100 mmHg) without signs of renal disease (GFR >60 mL/min/1.73 m^2^, absence of proteinuria, and normal renal ultrasound morphology).

In both groups, essential hypertension was diagnosed after having excluded secondary causes by clinical, biochemical, and imaging exams. All patients with diabetes mellitus, as defined by the American Diabetes Association were excluded from the study. After the initial screening visit at our Clinics, and before blood sampling, all the subjects selected were asked to sign an informed consent form for participating in this study, whose protocol had been previously approved by the Institutional Ethics Committee of the *Azienda Provinciale per Servizi Sanitari ASS1* (87.01/GEN/II.2/C8 issued on 20/02/2013).

#### General and biochemical parameters

After having given their informed consent, all the patients selected underwent a medical examination. Past medical history, the prevalence of macrovascular complications (including acute myocardial infarction, peripheral artery disease, and stroke), and medication were recorded. Office blood pressure was measured three times under antihypertensive therapy. In addition to that, all the patients underwent blood sampling for biochemical analyses, as well as a 24-h urinary collection. Samples were collected at 08.00 a.m., after an overnight fasting, as a part of our regular CKD patient follow-up, and they were centrifuged and stored at −80°. Creatinine, urea, uric acid, electrolytes, parathormone (PTH), glucose, triglycerides, total cholesterol, HDL cholesterol, and C-reactive protein (CRP) were measured by autoanalyzer. LDL cholesterol was calculated by the Friedwald’s formula. The GFR was calculated with the Cockcroft and Gault formula. Proteinuria was assessed by measurement of 24-h total urinary protein excretion with the Coomassie dye binding technique. The urinary albumin excretion rate (AER) was measured by a nephelometric method, as previously reported [30]. OPG was measured by ELISA in the sera (R&D; Cat#DY805).

### Experimental study

#### Study protocol

In order to evaluate whether an OPG increase could damage the kidney, we studied the gene and protein expression of pro-oxidative, proinflammatory, and profibrotic molecules in mice randomized to be treated with saline or OPG. Based on the protocol of one of our previous experimental studies [20], 18 adult (8-wk-old) male C57BL/6J mice (Harlan Laboratories, Udine, Italy) were randomized to receive human recombinant full-length OPG (OPG, *n* = 9) or saline (CNT, *n* = 9) every 3 weeks for 12 weeks. OPG (R&D Systems, Minneapolis) was delivered intraperitoneally at a dose of 1 μg per mouse in a total of 200 μl of saline. During the study period, all the mice were fed with a standard diet. The animals were kept in a temperature-controlled room (22 +/− 1 °C) on a 12 h light/ 12 h dark cycle with free access to food and water and they were fed ad libitum for the length of the study. After 12 weeks, the animals were anesthetized with an intraperitoneal injection of pentobarbital sodium (60 mg/kg body weight), and sacrificed by exanguination via cardiac puncture. Bloods and tissues were collected for further analyses. Animals were housed at the Animal House of the University of Trieste, and the protocol of this study was approved by its Animal Ethic Committee (ID 28.0.2008) in compliance with current guidelines on laboratory animal care and specific laws.

#### Gene expression analysis

Kidneys were divided and half of each was fixed in formalin, half in liquid nitrogen. The part that was snap-frozen in liquid nitrogen was used for gene expression analyses. Gene expression was measured by real-time quantitative RT-PCR (reverse transcription-polymerase chain reaction), as previously described [31]. Briefly, mRNA was extracted and then treated with the DNase inactivation reagent (Ambion DNA-free product #AM-1906), and 3 μg of treated mRNA were subsequently used to synthesize cDNA with Superscript First Strand synthesis system for RT-PCR (Gibco BRL). Gene expression was analysed by real-time quantitative RT-PCR using the TaqMan system (Life Technologies) for ACE, ACE2, AT1R, MCP-1, CTGF, and fibronectin, and the SYBR Green system (Life Technologies) for IL-6, TNF-α, TGF-β. Fluorescence for each cycle was quantitatively analysed by an ABI Prism 7900HT Sequence Detection System (Applied Biosystems). Gene expression of ACE, ACE2, AT1, MCP-1, CTGF, and fibronectin was normalized to 18S mRNA, while that of IL-6, TNF-α, TGF-β was normalized to Rps9. Results are reported as fold induction compared with the level of expression in untreated controls, which were given an arbitrary value of 1.

#### Immunostainings

The half of the kidney that was fixed in formalin, was embedded in paraffin, cut in 4-μm thick sections, and immunostained in order to measure the amount of glomerular nitrosylated protein. Kidney sections were incubated with rabbit anti-nitrotyrosine (Upstate, Lake Placid, NY; dilution 1:100) and biotinylated immunoglobulins (Vector laboratories, Burlingame, CA, dilution 1:500) which were then applied as secondary antibodies. Quantification of nitrotyrosine was performed by calculating the proportion of area occupied by the specific brown staining within the whole area of the glomerulus. For this analysis we used an image analysis system (Image-Pro Plus vers.6.3 Software, Media-Cybernetics; Silver Spring, MD, USA) associated with a high-resolution video-camera (Q-Imaging Fast 1394), and a computer.

### Statistical analysis

Data are presented as means ± SD. Significance was set at *p* < 0.05. The Kolmogorov-Smirnov test was applied to continuous variables to check for distribution normality. Cases and controls were compared using either the t-test for independent samples or the Mann-Whitney U test, where appropriate. The Pearson coefficient, for normally distributed variables, and the Spearman rank correlation coefficient, when at least one variable was not assumed to be normally distributed, were calculated to evaluate the correlation between OPG and age, sex, BMI, SBP, DBP, GFR, calcium, phosphate, PTH, and CRP once at a time. To evaluate the association of high circulating levels of OPG with CKD controlled for variables initially associated with CKD in the bivariate analyses, we tested a combination of multiple logistic regression models. Then, a receiver-operating characteristic (ROC) curve was designed to identify a cut-off value of OPG that best predicted the presence of CKD. Specificity and sensitivity were also calculated (95% confidence interval, CI). The best possible cut-off point was defined as the highest Youden Index [(specificity + sensibility) - 1], as previously reported [32]. Statistical analysis was performed with SAS 9.3 (SAS Institute, Cary, NC, USA). For the animal studies, results are expressed as means ± SD of the mean. Differences in the mean among groups were analysed using the t-test. Significance was set at *p* < 0.05.

## Results

### Patient characteristics

Baseline characteristics and laboratory data of CNT and CKD patients are reported in Table 1. As expected, hypertensive patients with CKD had lower BMI, higher SBP, and a higher prevalence of macrovascular events, as compared to controls. In addition, hypertensive patients with CKD had lower GFR, higher urea nitrogen, higher acid uric, and higher PTH, and they had proteinuria.

**Table 1:** Patient characteristics

Parameter	CNT (*n* = 141)	CKD (*n* = 42)	*p-value*
Age (years)	59.5 ± 7.7	60.1 ± 8.5	0.6467
Sex (% male)	68.1	5.7	0.0253
BMI (Kg/m^2^)	28.0 ± 4.6	25.4 ± 3.4	0.0002
SBP (mmHg)	155.2 ± 18.3	167.7 ± 21.6	0.0003
DBP (mmHg)	92.3 ± 9.2	94.9 ± 15.3	0.2987
Hypertension duration (months)	193 ± 94	212 ± 104	0.4558
Creatinine (mg/dL)	1.0 ± 0.1	3.4 ± 1.6	0.0001
GFR (mL/min)	69.0 ± 12.9	24.0 ± 13.1	<.0001
Urea nitrogen (mg/dL)	36.6 ± 9.3	94.9 ± 64.0	<.0001
Uric acid (mg/dL)	5.4 ± 1.4	7.1 ± 1.8	<.0001
Proteinuria (g/24 h)	-	1.3 ± 1.1	
AER (μg/min)	22.3 ± 45.0	-	
Glucose (mg/dL)	98.9 ± 1.7	93.4 ± 2.9	0.1188
Sodium (mEq/L)	140.6 ± 2.4	141.1 ± 2.8	0.2772
Potassium (mEq/L)	4.2 ± 0.3	4.6 ± 0.5	<.0001
Calcium (mg/dL)	5.2 ± 0.5	5.2 ± 0.8	0.9838
Phosphate (mg/dL)	2.8 ± 0.6	3.6 ± 1.5	0.0019
PTH (pg/mL)	33.7 ± 16.8	114.2 ± 110.6	<.0001
Total cholesterol (mg/dL)	235.3 ± 44.0	219.9 ± 50.8	0.0563
HDL cholesterol (mg/dL)	51.9 ± 15.0	44.2 ± 13.2	0.0032
Triglycerides (mg/dL)	148.1 ± 90.6	177.6 ± 69.1	0.2840
LDL cholesterol (mg/dL)	153.7 ± 40.2	140.1 ± 46.0	0.0892
CRP (mg/L)	3.9 ± 7.8	9.3 ± 11.31	0.0044
ACEi or/ARB (%)	52.5	35.7	0.0563
Macrovascular events (%)	12.8	54.8	<.0001

*ACEi* is for ACE inhibitors, *AER* is for albumin excretion rate, *ARB* is for AngiotensinII receptor blockers, *BMI* is for body mass index, *CKD* is for chronic kidney disease, *CNT* is for control, *CRP* is for C-reactive protein, *DBP* is for diastolic blood pressure, *GFR* is for glomerular filtration rate, *HDL* is for high density lipoprotein, *LDL* is for low density lipoprotein, *PTH* is for parathormone, *SBP* is for systolic blood pressure. Macrovascular events include acute myocardial infarction, peripheral artery disease, and stroke.

### OPG and renal function

OPG was significantly increased in CKD patients as compared to controls, being 2789.54 ± 2432.25 pg/mL in the CKD and 1216.18 ± 443.93 pg/mL in the CNT group (Satterthwaite t-test of means for unequal variances = 4.61, d.f. = 47.169; *p* < 0.001), for further details see the Additional file 1. Patients with CKD were further stratified into 3 groups according to National Kidney Foundation criteria for CKD: GFR 30–59 mL/min per 1.73 m^2^ (CKD3, *n* = 14), GFR 15–29 mL/min per 1.73 m^2^ (CKD4, *n* = 13), and GFR < 15 mL/min per 1.73 m^2^ (CKD5, *n* = 15) (Table 2). We found a continuous (and significant) increase of SBP, phosphate, PTH, CRP and OPG concentrations across the various CKD stages (Table 2). Interestingly, OPG and PTH were the only two parameters to increase significantly in patients with stage 3 CKD, whereas SBP, phosphate and CRP increased in patients with stage 4 CKD.

**Table 2:** Clinical and laboratory data stratified according to GFR in CKD stages

Parameter	CNT	Patients with CKD		
		CKD3 (*n* = 14)	CKD4 (*n* = 13)	CKD5 (*n* = 15)
SBP (mmHg)	155.25 ± 18.28	161.79 ± 20.15	168.46 ± 17.25	172.67 ± 25.97*
DBP (mmHg)	92.26 ± 9.20	93.21 ± 13.95	96.92 ± 18.09	94.67 ± 14.94
Calcium (mg/dL)	10.20 ± 0.54	10.09 ± 0.76	10.21 ± 0.71	10.30 ± 0.89
Phosphate (mg/dL)	2.76 ± 0.58	2.71 ± 0.92	3.55 ± 0.97*	4.41 ± 1.86*
PTH (pg/mL)	33.70 ± 16.7	57.00 ± 22.61*	126.37 ± 70.32*	160.89 ± 161.23*
CRP (mg/L)	4.03 ± 8.51	5.92 ± 4.83	6.83 ± 4.58*	15.9 ± 17.89*
OPG (pg/mL)	1216.18 ± 443.93	1619.11 ± 736.13*	1909.28 ± 1560.04*	4413.64 ± 3208.59*

*CKD* is for chronic kidney disease, *CNT* is for control, *CRP* is for C-reactive protein, *DBP* is for diastolic blood pressure, *OPG* is for osteoprotegerin, *PTH* is for parathormone, *SBP* is for systolic blood pressure. *p < 0.05

### OPG is inversely associated with renal function impairment

**Fig. 1:**
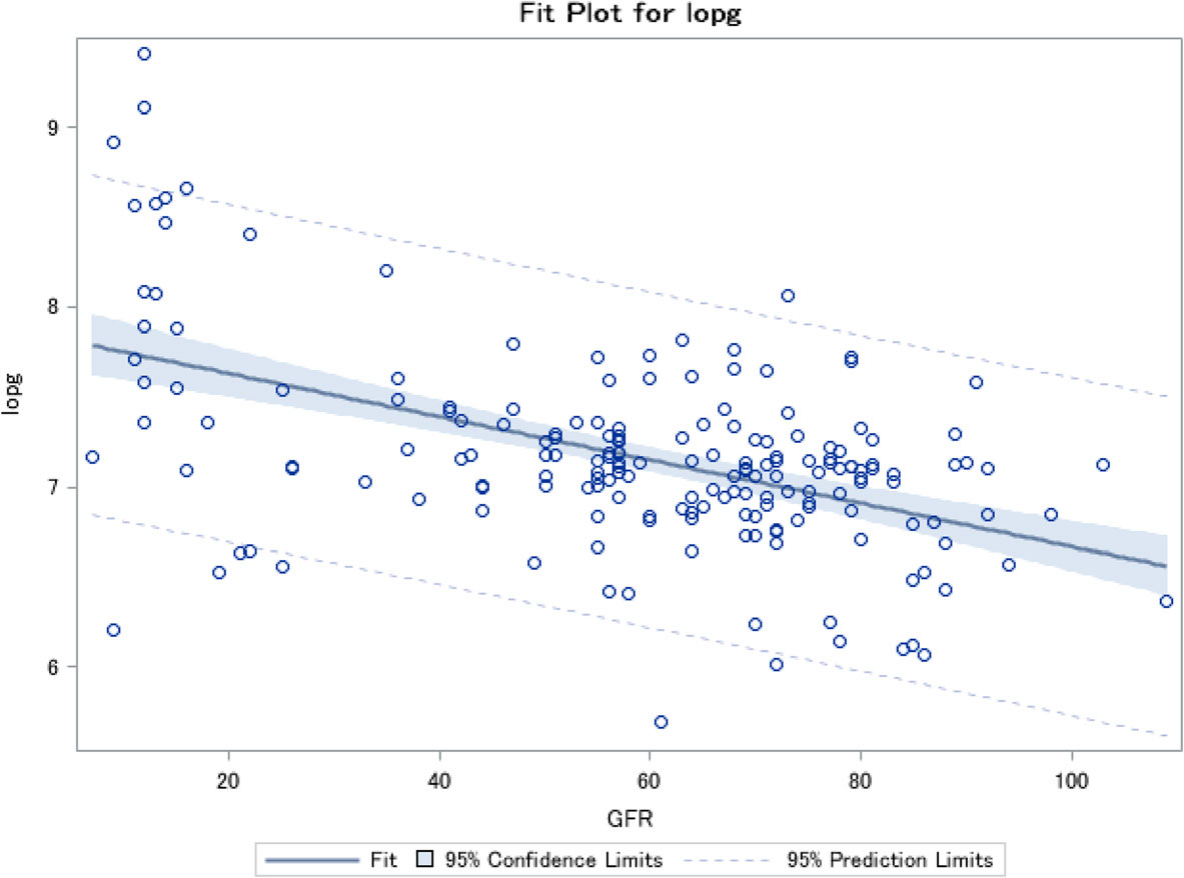
Inverse correlation between log OPG and GFR. Correlation between Log OPG (lopg) and GFR (Spearman coefficient = −0.40808 and *p* < 0.0001)

The univariate analysis showed that there was a significant inverse correlation between OPG and renal function (Fig. 1). Moreover, OPG was directly correlated with SBP (*p* < 0.0001), hypertension duration (*p* = 0.0018), PTH (*p* < 0.0001), phosphate (*p* < 0.0001), CRP (*p* < 0.0001), and macrovascular events (*p* < 0.0001). The multivariate analysis showed that OPG was significantly associated with CKD independently from age, sex, BMI, SBP, phosphate, PTH, and CRP (Table 3).

**Table 3:** Association of OPG with renal impairment from a mutually adjusted multiple logistic regression model^a^

Dependent variable: Renal impairment (yes/no)					
Predictive variables	DF	β estimate	Standard Error	Wald Chi-square	*p*-value
Log OPG	1	−2.1005	0.8004	6.8873	0.0087
BMI	1	0.2151	0.0880	5.9682	0.0146
SBP	1	−0.0178	0.0146	1.4779	0.2241
Col LDL	1	0.00910	0.00725	1.5737	0.2097
PTH	1	−0.0634	0.0155	16.6892	<0.0001
Phosphate	1	−0.6598	0.4620	2.0396	0.1532
CRP	1	0.000250	0.0254	0.0001	0.9921
Model R-square = 0.4306					

*BMI* is for body mass index, *CRP* is for C-reactive protein, *Col* LDL is for low density lipoprotein cholesterol, *OPG* is for osteoprotegerin, *PTH* is for parathormone, *SBP* is for systolic blood pressure

^a^Also adjusted for age and sex.

### OPG and CKD according to the area under the ROC curve

**Fig. 2:**
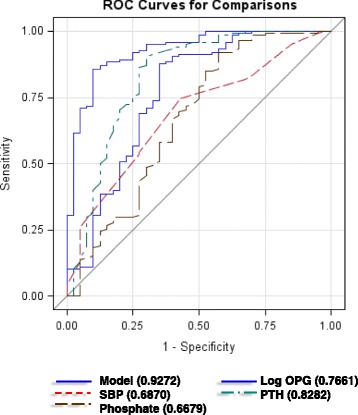
Predictive variables of CKD shown as ROC curves. Multivariate model of CKD as the dependent variable and Log OPG (blue line), SBP (red line), PTH (green line), phosphate (brown line), age, and sex as the explanatory variables

To evaluate the operating characteristics of OPG as a prognostic tool for the development of CKD in hypertensive patients, we performed a ROCanalysis for OPG with respect to CKD (Fig. 2). OPG was compared to BMI, phosphate, PTH, and SBP. Interestingly, OPG and PTH showed the highest predictive value for CKD development in hypertensive patients, as they obtained the highest areas under the curve (AUC), followed by phosphate and SBP. In particular, OPG had an AUC of 0.7661 and its cut-off value best predicting CKD was 1109.19 pg/mL (sensitivity = 85.8%; specificity = 59.5%), according to the maximum of the Youden Index.

### OPG delivery and kidney injury in vivo

**Fig. 3:**
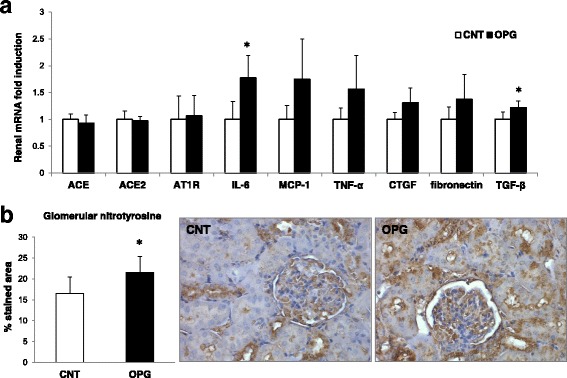
Effect of OPG delivery on renal injury. **a** Renal mRNA expression is reported as relative gene units; data is expressed as mean ± SD. *p < 0.05; ACE is for angiotensin-converting enzyme; ACE2 is for angiotensin-converting enzyme 2; AT1R is for angiotensinII type 1 receptor; IL-6 is for interleukin-6; MCP-1 is for monocyte chemoattractant protein-1; TNF-α is for tumor necrosis factor-α; CTGF is for connective tissue growth factor; TGF-β is for transforming growth factor-β (**b**) Semi-quantitative analysis of protein nitrosylation in the glomeruli, expressed as percentage stained area and representative sections of glomeruli stained for nitrotyrosine (original magnification 20X). Data is expressed as mean ± SD.﻿﻿ *p < 0.05

As it remains unclear whether OPG should be considered not only a risk marker but also a risk factor for CKD development, we evaluated the renal effects of repeated OPG injections in C57Bl/6J mice. In particular, based on the current understanding of the mechanisms/mediators underlying kidney disease [33], we studied the renal expression of TGF-β, which is considered the “master regulator” of glomerular and tubulointerstitial fibrosis [34]. In addition, among the ever-growing panel of molecules that are known to elicit fibrosis-promoting effects and effects on the pathway of TGF-β [33], we studied the renal expression of members of the renin-angiotensin-aldosterone system (ACE, ACE2, AT1R), other growth factors (CTGF, TNF-α), cytokines (IL-6), chemokines (MCP-1), matrix molecules (fibronectin), as well as the renal nitrosylation of proteins, which is a marker of oxidative stress, and renal damage. We found that OPG delivery was associated with a significant increase in the gene expression of IL-6 and TGF-β and with a significant increase in protein nitrosylation (Fig. 3), for further details see the Additional file 1.

## Discussion

This study shows that circulating OPG is significantly associated with the presence of CKD in hypertensive non-diabetic patients, independently from other variables. This is consistent with the report that plasma OPG concentration predicts the deterioration of kidney function in type 1 diabetic patients [25], and suggests that OPG could be used as a risk marker for CKD not only in type 1 diabetes, but also in patients with hypertension, as it was shown in elderly women [27] and renal transplant recipients [35]. In this study, although systolic blood pressure was significantly different between the groups and was conceivably the underlying cause of CKD, we found that the association between OPG and CKD was independent from blood pressure, and that, quite interestingly, OPG increased at an earlier stage of CKD as compared to hypertension.

In the ROC analysis, OPG displayed a high AUC for CKD, and the cut-off value best predicting CKD was in line with the values reported by Bjerre [36]. Interestingly, OPG turned out to be associated with a larger AUC than that of hypertension, but similar to that of PTH. Classically, OPG and PTH are both related to bone metabolism [37, 38]. Nevertheless, the fact that OPG and PTH were both increased in CKD and that they had a similar predictive value on CKD status should not be due to a direct stimulatory effect of PTH on OPG. It has in fact been demonstrated that PTH downregulates OPG expression in bone cells [39–41], and that, on the other hand, OPG does not change PTH levels [42]. Moreover, in this study, the association between OPG and CKD was independent from PTH. It is actually possible that the similar behaviour of OPG and PTH relies on a shared trigger, such as the renin-angiotensin-aldosterone system (RAAS) activation, for example [43, 44]. It has been demonstrated that RAAS activation might increase OPG levels, which is supported by the experimental observation that AngII increased OPG expression in aortic smooth muscle cells in vitro [45, 46].

Other mechanisms underlying OPG increase in CKD might include low-grade inflammation, FGF-23 elevation, and kidney function itself. Inflammation might contribute to elevate OPG levels as several proinflammatory cytokines, such as TNF-α, regulate OPG production in vascular smooth muscle cells [47]. With respect to this point, we found a significant association between OPG and CRP, not only in the hypertensive patients selected for this study, but also in patients with metabolic syndrome that we selected for a previous study [48]. Secondly, elevated FGF-23 levels which are found in CKD patients, might also stimulate OPG expression [49]. Thirdly, given that kidney excretion is supposed to regulate the clearance of OPG, the retention of OPG due to renal impairment might provide another part of the answer to the mechanisms whereby OPG concentrations are increased in CKD [26].

Beside the mechanisms causing OPG elevation, it remains to be answered what are OPG effects on the kidney. Several works have demonstrated that OPG is not just a marker but also a risk factor of disease [20], as its delivery induced proinflammatory and profibrotic tissue changes at different levels [20, 45, 48, 50]. As for inflammation, it has been demonstrated that OPG stimulated endothelial cell expression of adhesion molecules [51], and that it increased leukocyte adhesion to endothelial cells [52]. As for fibrosis, we have recently shown that OPG was able to initiate TGF-β1-dependent changes in vascular smooth muscle cells [45], whereby it stimulated proliferation, inflammation, and fibrosis. Moreover, also OPG expression increased in response to TGF-β1, which could lead to a vicious cycle that results in the auto-induction of both OPG and TGF-β1.

Consistent with these experimental observations, here we found that OPG delivery significantly increased the gene expression of IL-6 and TGF-β, as well as the amount of protein nitrosylation in the kidney, which are all involved in kidney damage development and progression. Animal studies have established that most renal diseases (including hypertensive CKD) progress to renal failure as a consequence of functional adaptations which ultimately lead to fibrosis [53]. In this process, not only TGF-β, but also several cytokines, growth factors, and vasoactive substances promote the abnormal accumulation of extracellular matrix collagen, fibronectin, and other components that are responsible for renal scarring [33]. Therefore, our data suggests that OPG has the potential to directly induce kidney damage, as it significantly upregulated TGF-β, which is considered the primary factor that drives fibrosis in most forms of CKD [34]. In addition, also OPG-induced IL-6 upregulation and oxidative stress show that OPG can directly damage the kidney, as it has been shown that IL-6 expression increases in diabetic nephropathy [54], and that oxidative stress induces fibrogenesis in CKD [55]. Unexpectedly, we did not find any change in the gene expression of RAAS components. This might have been due to our treatment protocol. Further studies with different treatment schedules will help characterize more in detail OPG effects on the kidney.

## Conclusions

This study shows that circulating OPG was significantly associated with the presence of CKD in hypertensive non-diabetic patients, independently from other variables. This suggests that OPG could be a risk marker for hypertension-induced CKD. In addition, in the experimental study, OPG delivery caused molecular changes associated with kidney damage, which sheds light on OPG potential to be not only a risk marker but also a risk factor for CKD.

There are a few limitations in the clinical study. Our findings refer to a Caucasian population. In the group of patients with CKD there were more women, however, this should not have affected our final data because it has been demonstrated that serum OPG levels are not different in post-menopausal women compared to men [56], and our multivariate analyses were adjusted for age and sex. The sample size is relatively small. The case-control design does not allow determining whether OPG, which was significantly associated with CKD development, can actually predict CKD development. This requires further prospective studies, such as the Tromsø study [15]. With respect to our experimental data, it is possible that OPG-treated mice did not show any change in the RAAS expression because of our experimental protocol.

Therefore, further studies are needed not only to evaluate whether OPG predicts CKD development, but also to support the notion that OPG is a risk factor for kidney disease.

## Additional file

Additional file 1: 1. Patient OPG and GFR values. Raw data of patient circulating OPG values (pg/mL) as assessed by ELISA matched with GFR values (ml/min). 2. Gene expression analysis. Raw data of kidney mRNA expression of ACE, ACE2, AT1R, MCP-1, CTGF, and fibronectin, IL-6, TNF-α, TGF-β. 3. Glomerular nitrotyrosine staining. Raw data of semi-quantitative analysis of protein nitrosylation in the glomeruli, expressed as percentage stained area (brown)/area glomerulus. (DOCX 64 kb)

## Abbreviations

ACE: angiotensin-converting enzyme;
ACE2: angiotensin-converting enzyme-2
AER: albumin excretion rate
AT1R: angiotensin II type 1 receptor
AUC: area under the curve
BMI: body mass index
CKD: chronic kidney disease
CRP: C-reactive protein
CTGF: connective tissue growth factor
CVD: cardiovascular diseases
DBP: diastolic blood pressure
ELISA: enzyme-linked immunosorbent assay
FGF-23: fibroblast growth factor-23
GFR: glomerular filtration rate
HDL: high-density lipoprotein
IL-6: interleukin-6
LDL: low-density lipoprotein
MCP-1: monocyte chemoattractant protein-1
OPG: osteoprotegerin
PTH: parathormone
RAAS: renin-angiotensin-aldosterone system
RANKL: receptor activator for nuclear factor kB ligand
ROC: receiving-operating characteristic
RT-PCR: reverse transcription-polymerase chain reaction
SBP: systolic blood pressure
TGF-β: transforming growth factor- β
TNF-α: tumor necrosis factor-α
TRAIL: TNF-related apoptosis-inducing ligand

## References

[1] Vitovski S, Phillips JS, Sayers J, Croucher PI (2007). Investigating the interaction between osteoprotegerin and receptor activator of NF-kappaB or tumor necrosis factor-related apoptosis-inducing ligand: evidence for a pivotal role for osteoprotegerin in regulating two distinct pathways. J Biol Chem.

[2] American Society for B, Mineral Research President's Committee on N: Proposed standard nomenclature for new tumor necrosis factor family members involved in the regulation of bone resorption. The American Society for Bone and Mineral Research President's Committee on Nomenclature. *J Bone Miner Res* 2000, **15**(12):2293–2296. 10.1359/jbmr.2000.15.12.2293 11127193

[3] Hofbauer LC, Schoppet M (2004). Clinical implications of the osteoprotegerin/RANKL/RANK system for bone and vascular diseases. JAMA.

[4] Venuraju SM, Yerramasu A, Corder R, Lahiri A (2010). Osteoprotegerin as a predictor of coronary artery disease and cardiovascular mortality and morbidity. J Am Coll Cardiol.

[5] Ziegler S, Kudlacek S, Luger A, Minar E (2005). Osteoprotegerin plasma concentrations correlate with severity of peripheral artery disease. Atherosclerosis.

[6] Shin JY, Shin YG, Chung CH (2006). Elevated serum osteoprotegerin levels are associated with vascular endothelial dysfunction in type 2 diabetes. Diabetes Care.

[7] Zagura M, Serg M, Kampus P, Zilmer M, Zilmer K, Eha J (2010). Association of osteoprotegerin with aortic stiffness in patients with symptomatic peripheral artery disease and in healthy subjects. Am J Hypertens.

[8] Abedin M, Omland T, Ueland T, Khera A, Aukrust P, Murphy SA (2007). Relation of osteoprotegerin to coronary calcium and aortic plaque (from the Dallas heart study). Am J Cardiol.

[9] Jono S, Ikari Y, Shioi A, Mori K, Miki T, Hara K (2002). Serum osteoprotegerin levels are associated with the presence and severity of coronary artery disease. Circulation.

[10] Schoppet M, Sattler AM, Schaefer JR, Herzum M, Maisch B, Hofbauer LC (2003). Increased osteoprotegerin serum levels in men with coronary artery disease. J Clin Endocrinol Metab.

[11] Semb AG, Ueland T, Aukrust P, Wareham NJ, Luben R, Gullestad L (2009). Osteoprotegerin and soluble receptor activator of nuclear factor-kappaB ligand and risk for coronary events: a nested case-control approach in the prospective EPIC-Norfolk population study 1993-2003. Arterioscler Thromb Vasc Biol.

[12] Kiechl S, Schett G, Wenning G, Redlich K, Oberhollenzer M, Mayr A (2004). Osteoprotegerin is a risk factor for progressive atherosclerosis and cardiovascular disease. Circulation.

[13] Omland T, Ueland T, Jansson AM, Persson A, Karlsson T, Smith C (2008). Circulating osteoprotegerin levels and long-term prognosis in patients with acute coronary syndromes. J Am Coll Cardiol.

[14] Jono S, Otsuki S, Higashikuni Y, Shioi A, Mori K, Hara K (2010). Serum osteoprotegerin levels and long-term prognosis in subjects with stable coronary artery disease. J Thromb Haemost.

[15] Vik A, Mathiesen EB, Brox J, Wilsgaard T, Njolstad I, Jorgensen L (2011). Serum osteoprotegerin is a predictor for incident cardiovascular disease and mortality in a general population: the Tromso study. J Thromb Haemost.

[16] Ueland T, Jemtland R, Godang K, Kjekshus J, Hognestad A, Omland T (2004). Prognostic value of osteoprotegerin in heart failure after acute myocardial infarction. J Am Coll Cardiol.

[17] Lieb W, Gona P, Larson MG, Massaro JM, Lipinska I, Keaney JF (2010). Biomarkers of the osteoprotegerin pathway: clinical correlates, subclinical disease, incident cardiovascular disease, and mortality. Arterioscler Thromb Vasc Biol.

[18] Bucay N, Sarosi I, Dunstan CR, Morony S, Tarpley J, Capparelli C (1998). Lacey DL *et al*: osteoprotegerin-deficient mice develop early onset osteoporosis and arterial calcification. Genes Dev.

[19] Bennett BJ, Scatena M, Kirk EA, Rattazzi M, Varon RM, Averill M (2006). Osteoprotegerin inactivation accelerates advanced atherosclerotic lesion progression and calcification in older ApoE−/− mice. Arterioscler Thromb Vasc Biol.

[20] Candido R, Toffoli B, Corallini F, Bernardi S, Zella D, Voltan R (2010). Human full-length osteoprotegerin induces the proliferation of rodent vascular smooth muscle cells both in vitro and in vivo. J Vasc Res.

[21] Bernardi S, Bossi F, Toffoli B, Fabris B (2016). Roles and clinical applications of OPG and TRAIL as biomarkers in cardiovascular disease. Biomed Res Int.

[22] Kazama JJ, Shigematsu T, Yano K, Tsuda E, Miura M, Iwasaki Y (2002). Increased circulating levels of osteoclastogenesis inhibitory factor (osteoprotegerin) in patients with chronic renal failure. Am J Kidney Dis.

[23] Upadhyay A, Larson MG, Guo CY, Vasan RS, Lipinska I, O'Donnell CJ (2011). Inflammation, kidney function and albuminuria in the Framingham offspring cohort. Nephrol Dial Transplant.

[24] Rasmussen LM, Tarnow L, Hansen TK, Parving HH, Flyvbjerg A (2006). Plasma osteoprotegerin levels are associated with glycaemic status, systolic blood pressure, kidney function and cardiovascular morbidity in type 1 diabetic patients. Eur J Endocrinol.

[25] Jorsal A, Tarnow L, Flyvbjerg A, Parving HH, Rossing P, Rasmussen LM (2008). Plasma osteoprotegerin levels predict cardiovascular and all-cause mortality and deterioration of kidney function in type 1 diabetic patients with nephropathy. Diabetologia.

[26] Gordin D, Soro-Paavonen A, Thomas MC, Harjutsalo V, Saraheimo M, Bjerre M (2013). Osteoprotegerin is an independent predictor of vascular events in Finnish adults with type 1 diabetes. Diabetes Care.

[27] Lewis JR, Lim WH, Zhu K, Wong G, Dhaliwal SS, Lim EM (2014). Elevated osteoprotegerin predicts declining renal function in elderly women: a 10-year prospective cohort study. Am J Nephrol.

[28] Montanez-Barragan A, Gomez-Barrera I, Sanchez-Nino MD, Ucero AC, Gonzalez-Espinoza L, Ortiz A. Osteoprotegerin and kidney disease. J Nephrol. 2014; 10.1007/s40620-014-0092-x 24756971

[29] Candido R (2014). The osteoprotegerin/tumor necrosis factor related apoptosis-inducing ligand axis in the kidney. Curr Opin Nephrol Hypertens.

[30] Fabris B, Bortoletto M, Candido R, Barbone F, Cattin MR, Calci M (2005). Genetic polymorphisms of the renin-angiotensin-aldosterone system and renal insufficiency in essential hypertension. J Hypertens.

[31] Bernardi S, Tikellis C, Candido R, Tsorotes D, Pickering RJ, Bossi F (2015). ACE2 deficiency shifts energy metabolism towards glucose utilization. Metabolism.

[32] Morena M, Dupuy AM, Jaussent I, Vernhet H, Gahide G, Klouche K (2009). A cut-off value of plasma osteoprotegerin level may predict the presence of coronary artery calcifications in chronic kidney disease patients. Nephrol Dial Transplant.

[33] Eddy AA: Overview of the cellular and molecular basis of kidney fibrosis. *Kidney Int Suppl (2011)*. 2014, 4(1):2–8. 10.1038/kisup.2014.2 PMC4220516 25401038

[34] Meng XM, Nikolic-Paterson DJ, Lan HY (2016). TGF-beta: the master regulator of fibrosis. Nat Rev Nephrol.

[35] Svensson M, Dahle DO, Mjoen G, Weihrauch G, Scharnagl H, Dobnig H (2012). Osteoprotegerin as a predictor of renal and cardiovascular outcomes in renal transplant recipients: follow-up data from the ALERT study. Nephrol Dial Transplant.

[36] Bjerre M (2013). Osteoprotegerin (OPG) as a biomarker for diabetic cardiovascular complications. Spring.

[37] Kwon BS, Wang S, Udagawa N, Haridas V, Lee ZH, Kim KK (1998). TR1, a new member of the tumor necrosis factor receptor superfamily, induces fibroblast proliferation and inhibits osteoclastogenesis and bone resorption. FASEB J.

[38] Silva BC, Bilezikian JP (2015). Parathyroid hormone: anabolic and catabolic actions on the skeleton. Curr Opin Pharmacol.

[39] Kanzawa M, Sugimoto T, Kanatani M, Chihara K (2000). Involvement of osteoprotegerin/osteoclastogenesis inhibitory factor in the stimulation of osteoclast formation by parathyroid hormone in mouse bone cells. Eur J Endocrinol.

[40] Huang JC, Sakata T, Pfleger LL, Bencsik M, Halloran BP, Bikle DD (2004). PTH differentially regulates expression of RANKL and OPG. J Bone Miner Res.

[41] Onyia JE, Miles RR, Yang X, Halladay DL, Hale J, Glasebrook A (2000). In vivo demonstration that human parathyroid hormone 1-38 inhibits the expression of osteoprotegerin in bone with the kinetics of an immediate early gene. J Bone Miner Res.

[42] Bone HG, Bolognese MA, Yuen CK, Kendler DL, Miller PD, Yang YC (2011). Effects of denosumab treatment and discontinuation on bone mineral density and bone turnover markers in postmenopausal women with low bone mass. J Clin Endocrinol Metab.

[43] Brown JM, Williams JS, Luther JM, Garg R, Garza AE, Pojoga LH (2014). Human interventions to characterize novel relationships between the renin-angiotensin-aldosterone system and parathyroid hormone. Hypertension.

[44] Koiwa F, Komukai D, Hirose M, Yoshimura A, Ando R, Sakaguchi T (2012). Influence of renin-angiotensin system on serum parathyroid hormone levels in uremic patients. Clin Exp Nephrol.

[45] Toffoli B, Pickering RJ, Tsorotes D, Wang B, Bernardi S, Kantharidis P (2011). Osteoprotegerin promotes vascular fibrosis via a TGF-beta1 autocrine loop. Atherosclerosis.

[46] Zhang J, Fu M, Myles D, Zhu X, Du J, Cao X (2002). PDGF induces osteoprotegerin expression in vascular smooth muscle cells by multiple signal pathways. FEBS Lett.

[47] Olesen P, Ledet T, Rasmussen LM (2005). Arterial osteoprotegerin: increased amounts in diabetes and modifiable synthesis from vascular smooth muscle cells by insulin and TNF-alpha. Diabetologia.

[48] Bernardi S, Fabris B, Thomas M, Toffoli B, Tikellis C, Candido R (2014). Osteoprotegerin increases in metabolic syndrome and promotes adipose tissue proinflammatory changes. Mol Cell Endocrinol.

[49] Nakahara T, Kawai-Kowase K, Matsui H, Sunaga H, Utsugi T, Iso T (2016). Fibroblast growth factor 23 inhibits osteoblastic gene expression and induces osteoprotegerin in vascular smooth muscle cells. Atherosclerosis.

[50] Toffoli B, Bernardi S, Candido R, Sabato N, Carretta R, Corallini F (2011). Osteoprotegerin induces morphological and functional alterations in mouse pancreatic islets. Mol Cell Endocrinol.

[51] Mangan SH, Van Campenhout A, Rush C, Golledge J (2007). Osteoprotegerin upregulates endothelial cell adhesion molecule response to tumor necrosis factor-alpha associated with induction of angiopoietin-2. Cardiovasc Res.

[52] Zauli G, Corallini F, Bossi F, Fischetti F, Durigutto P, Celeghini C (2007). Osteoprotegerin increases leukocyte adhesion to endothelial cells both in vitro and in vivo. Blood.

[53] Cortinovis M, Ruggenenti P, Remuzzi G (2016). Progression, remission and regression of chronic renal diseases. Nephron.

[54] Suzuki D, Miyazaki M, Naka R, Koji T, Yagame M, Jinde K (1995). In situ hybridization of interleukin 6 in diabetic nephropathy. Diabetes.

[55] Okamura DM, Bahrami NM, Ren S, Pasichnyk K, Williams JM, Gangoiti JA (2014). Cysteamine modulates oxidative stress and blocks myofibroblast activity in CKD. J Am Soc Nephrol.

[56] Khosla S, Arrighi HM, Melton LJ, Atkinson EJ, O'Fallon WM, Dunstan C (2002). Correlates of osteoprotegerin levels in women and men. Osteoporos Int.

