# Supplementary material for: Circulating osteoprotegerin is associated with chronic kidney disease in hypertensive patients
Source: BMC Nephrol. 2017 Jul 6;18:219. doi: 10.1186/s12882-017-0625-3 (PMC5500921; doi:10.1186/s12882-017-0625-3)
Supplement: Additional file 1: — 1. Patient OPG and GFR values. Raw data of patient circulating OPG values (pg/mL) as assessed by ELISA matched with GFR values (ml/min). 2. Gene expression analysis. Raw data of kidney mRNA expression of ACE, ACE2, AT1R, MCP-1, CTGF, and fibronectin, IL-6, TNF-α, TGF-β. 3. Glomerular nitrotyrosine staining. Raw data of semi-quantitative analysis of protein nitrosylation in the glomeruli, expressed as percentage stained area (brown)/area glomerulus. (DOCX 64 kb) [file 12882_2017_625_MOESM1_ESM.docx]

**Additional file 1**

**1. Patient OPG and GFR values:**

| **GROUP** | **OPG pg/ml** | **GFR ml/min** |  | **GROUP** | **OPG pg/ml** | **GFR ml/min** |
| --- | --- | --- | --- | --- | --- | --- |
| **CKD** | 721,69 | 49 |  | **CNT** | 619,28 | 88 |
| **CKD** | 1101,97 | 44 |  | **CNT** | 2096,46 | 71 |
| **CKD** | 1666,08 | 41 |  | **CNT** | 296,12 | 61 |
| **CKD** | 1780,94 | 36 |  | **CNT** | 909,51 | 60 |
| **CKD** | 3239,85 | 12 |  | **CNT** | 1577,18 | 53 |
| **CKD** | 1213,43 | 26 |  | **CNT** | 854,31 | 72 |
| **CKD** | 3219,25 | 13 |  | **CNT** | 1166,72 | 58 |
| **CKD** | 9058,26 | 12 |  | **CNT** | 1419,50 | 70 |
| **CKD** | 1294,03 | 7 |  | **CNT** | 1217,45 | 69 |
| **CKD** | 1231,73 | 26 |  | **CNT** | 942,33 | 98 |
| **CKD** | 4767,28 | 14 |  | **CNT** | 1475,22 | 89 |
| **CKD** | 5261,88 | 11 |  | **CNT** | 2110,52 | 68 |
| **CKD** | 1714,13 | 41 |  | **CNT** | 1159,74 | 50 |
| **CKD** | 1700,33 | 47 |  | **CNT** | 1201,33 | 69 |
| **CKD** | 5775,27 | 16 |  | **CNT** | 1069,10 | 68 |
| **CKD** | 2221,78 | 11 |  | **CNT** | 1406,89 | 71 |
| **CKD** | 1884,09 | 25 |  | **CNT** | 1307,74 | 50 |
| **CKD** | 1313,33 | 43 |  | **CNT** | 1326,61 | 56 |
| **CKD** | 1023,20 | 38 |  | **CNT** | 976,16 | 65 |
| **CKD** | 1125,85 | 33 |  | **CNT** | 998,12 | 75 |
| **CKD** | 1964,11 | 12 |  | **CNT** | 919,33 | 64 |
| **CKD** | 1202,59 | 16 |  | **CNT** | 835,24 | 69 |
| **CKD** | 4470,12 | 22 |  | **CNT** | 1039,68 | 67 |
| **CKD** | 5320,38 | 13 |  | **CNT** | 818,02 | 80 |
| **CKD** | 2655,32 | 15 |  | **CNT** | 1593,61 | 42 |
| **CKD** | 1908,94 | 15 |  | **CNT** | 1270,38 | 64 |
| **CKD** | 2423,41 | 47 |  | **CNT** | 1304,73 | 51 |
| **CKD** | 1352,81 | 37 |  | **CNT** | 832,79 | 70 |
| **CKD** | 2004,55 | 36 |  | **CNT** | 1240,29 | 71 |
| **CKD** | 1096,23 | 44 |  | **CNT** | 1521,01 | 80 |
| **CKD** | 681,90 | 19 |  | **CNT** | 2019,47 | 64 |
| **CKD** | 1565,36 | 18 |  | **CNT** | 516,66 | 77 |
| **CKD** | 703,66 | 25 |  | **CNT** | 1246,85 | 103 |
| **CKD** | 1576,70 | 12 |  | **CNT** | 950,75 | 64 |
| **CKD** | 12215,76 | 12 |  | **CNT** | 1248,31 | 59 |
| **CKD** | 7437,04 | 9 |  | **CNT** | 444,07 | 84 |
| **CKD** | 2672,21 | 12 |  | **CNT** | 1291,49 | 72 |
| **CKD** | 5461,42 | 14 |  | **CNT** | 1063,08 | 73 |
| **CKD** | 494,71 | 9 |  | **CNT** | 1983,83 | 56 |
| **CKD** | 768,80 | 22 |  | **CNT** | 1031,77 | 64 |
| **CKD** | 759,45 | 21 |  | **CNT** | 464,53 | 78 |
| **CKD** | 3642,99 | 35 |  | **CNT** | 782,83 | 55 |
|  |  |  |  | **CNT** | 1292,90 | 56 |
|  |  |  |  | **CNT** | 509,91 | 70 |
|  |  |  |  | **CNT** | 891,89 | 85 |
|  |  |  |  | **CNT** | 2282,45 | 60 |
|  |  |  |  | **CNT** | 616,27 | 56 |
|  |  |  |  | **CNT** | 1149,79 | 55 |
|  |  |  |  | **CNT** | 2253,15 | 55 |
|  |  |  |  | **CNT** | 1419,86 | 81 |
|  |  |  |  | **CNT** | 2472,59 | 63 |
|  |  |  |  | **CNT** | 3188,10 | 73 |
|  |  |  |  | **CNT** | 1081,99 | 66 |
|  |  |  |  | **CNT** | 1449,83 | 56 |
|  |  |  |  | **CNT** | 1286,34 | 42 |
|  |  |  |  | **CNT** | 1141,29 | 56 |
|  |  |  |  | **CNT** | 1361,86 | 77 |
|  |  |  |  | **CNT** | 1420,80 | 57 |
|  |  |  |  | **CNT** | 1414,11 | 57 |
|  |  |  |  | **CNT** | 454,16 | 85 |
|  |  |  |  | **CNT** | 1536,53 | 68 |
|  |  |  |  | **CNT** | 1329,96 | 57 |
|  |  |  |  | **CNT** | 1406,90 | 50 |
|  |  |  |  | **CNT** | 1344,43 | 78 |
|  |  |  |  | **CNT** | 1108,76 | 55 |
|  |  |  |  | **CNT** | 715,75 | 94 |
|  |  |  |  | **CNT** | 1263,41 | 75 |
|  |  |  |  | **CNT** | 1131,62 | 83 |
|  |  |  |  | **CNT** | 932,72 | 55 |
|  |  |  |  | **CNT** | 1035,45 | 71 |
|  |  |  |  | **CNT** | 1959,58 | 91 |
|  |  |  |  | **CNT** | 1687,36 | 67 |
|  |  |  |  | **CNT** | 1553,86 | 65 |
|  |  |  |  | **CNT** | 1268,20 | 55 |
|  |  |  |  | **CNT** | 1997,18 | 60 |
|  |  |  |  | **CNT** | 2207,66 | 79 |
|  |  |  |  | **CNT** | 1567,99 | 55 |
|  |  |  |  | **CNT** | 1456,81 | 57 |
|  |  |  |  | **CNT** | 1438,08 | 51 |
|  |  |  |  | **CNT** | 1205,51 | 80 |
|  |  |  |  | **CNT** | 1427,57 | 57 |
|  |  |  |  | **CNT** | 1312,95 | 66 |
|  |  |  |  | **CNT** | 1160,27 | 72 |
|  |  |  |  | **CNT** | 1192,24 | 57 |
|  |  |  |  | **CNT** | 608,74 | 58 |
|  |  |  |  | **CNT** | 900,63 | 87 |
|  |  |  |  | **CNT** | 944,48 | 92 |
|  |  |  |  | **CNT** | 939,99 | 69 |
|  |  |  |  | **CNT** | 931,95 | 60 |
|  |  |  |  | **CNT** | 765,93 | 64 |
|  |  |  |  | **CNT** | 1171,19 | 83 |
|  |  |  |  | **CNT** | 1472,00 | 51 |
|  |  |  |  | **CNT** | 1518,80 | 57 |
|  |  |  |  | **CNT** | 1162,48 | 68 |
|  |  |  |  | **CNT** | 960,79 | 79 |
|  |  |  |  | **CNT** | 930,95 | 70 |
|  |  |  |  | **CNT** | 1032,62 | 57 |
|  |  |  |  | **CNT** | 861,95 | 72 |
|  |  |  |  | **CNT** | 1240,67 | 81 |
|  |  |  |  | **CNT** | 964,03 | 44 |
|  |  |  |  | **CNT** | 653,71 | 85 |
|  |  |  |  | **CNT** | 2253,53 | 79 |
|  |  |  |  | **CNT** | 980,94 | 75 |
|  |  |  |  | **CNT** | 1060,82 | 78 |
|  |  |  |  | **CNT** | 1147,74 | 80 |
|  |  |  |  | **CNT** | 431,77 | 86 |
|  |  |  |  | **CNT** | 968,89 | 63 |
|  |  |  |  | **CNT** | 987,48 | 71 |
|  |  |  |  | **CNT** | 583,17 | 109 |
|  |  |  |  | **CNT** | 1256,35 | 69 |
|  |  |  |  | **CNT** | 1067,66 | 75 |
|  |  |  |  | **CNT** | 1089,48 | 54 |
|  |  |  |  | **CNT** | 1226,50 | 79 |
|  |  |  |  | **CNT** | 1247,06 | 89 |
|  |  |  |  | **CNT** | 1251,12 | 90 |
|  |  |  |  | **CNT** | 1558,30 | 46 |
|  |  |  |  | **CNT** | 1107,62 | 50 |
|  |  |  |  | **CNT** | 797,72 | 88 |
|  |  |  |  | **CNT** | 906,32 | 74 |
|  |  |  |  | **CNT** | 1436,60 | 63 |
|  |  |  |  | **CNT** | 1280,06 | 77 |
|  |  |  |  | **CNT** | 1210,26 | 78 |
|  |  |  |  | **CNT** | 1321,20 | 57 |
|  |  |  |  | **CNT** | 1231,70 | 57 |
|  |  |  |  | **CNT** | 2362,78 | 68 |
|  |  |  |  | **CNT** | 1273,59 | 72 |
|  |  |  |  | **CNT** | 1251,24 | 77 |
|  |  |  |  | **CNT** | 802,31 | 72 |
|  |  |  |  | **CNT** | 1053,81 | 69 |
|  |  |  |  | **CNT** | 1162,18 | 70 |
|  |  |  |  | **CNT** | 1463,78 | 74 |
|  |  |  |  | **CNT** | 1229,17 | 79 |
|  |  |  |  | **CNT** | 1131,60 | 80 |
|  |  |  |  | **CNT** | 679,93 | 86 |
|  |  |  |  | **CNT** | 1218,09 | 81 |
|  |  |  |  | **CNT** | 1256,31 | 57 |
|  |  |  |  | **CNT** | 1211,97 | 92 |
|  |  |  |  | **CNT** | 1186,34 | 76 |
|  |  |  |  | **CNT** | 1658,26 | 73 |
|  |  |  |  | **CNT** | 408,98 | 72 |
|  |  |  |  | **CNT** | 1190,36 | 55 |

**2. Gene expression analysis:**

| **Prot OPG : Kidney ACE** | |  |  | TaqMan |  |  |  |  |  |
| --- | --- | --- | --- | --- | --- | --- | --- | --- | --- |
|  |  |  |  |  |  |  |  |  |  |
| **Treatment** | **FAM** | **VIC** | **dCt** | **Av cont.** | **dd ct** | **fold incr.** | **average** | **St.Dev** | **SEM** |
| CNT | 20,21 | 9,38 | 10,82 |  | -0,19 | 1,14 |  |  |  |
| CNT | 20,45 | 9,32 | 11,13 |  | 0,11 | 0,93 |  |  |  |
| CNT | 20,32 | 9,08 | 11,24 |  | 0,22 | 0,86 |  |  |  |
| CNT | 20,15 | 9,22 | 10,93 |  | -0,09 | 1,06 |  |  |  |
| CNT | 20,12 | 9,16 | 10,97 |  | -0,05 | 1,03 |  |  |  |
| CNT | 19,94 | 9,01 | 10,93 |  | -0,09 | 1,06 |  |  |  |
| CNT | 20,23 | 9,13 | 11,10 | **11,02** | 0,08 | 0,94 | **1,00** | **0,10** | **0,04** |
| OPG | 19,90 | 8,93 | 10,97 |  | -0,04 | 1,03 |  |  |  |
| OPG | 20,06 | 9,19 | 10,87 |  | -0,15 | 1,11 |  |  |  |
| OPG | 20,09 | 9,13 | 10,96 |  | -0,06 | 1,04 |  |  |  |
| OPG | 20,28 | 9,25 | 11,03 |  | 0,02 | 0,99 |  |  |  |
| OPG | 20,20 | 9,06 | 11,14 |  | 0,12 | 0,92 |  |  |  |
| OPG | 20,52 | 9,17 | 11,36 |  | 0,34 | 0,79 |  |  |  |
| OPG | 20,14 | 9,08 | 11,06 |  | 0,05 | 0,97 |  |  |  |
| OPG | 20,82 | 9,16 | 11,66 |  | 0,64 | 0,64 | **0,94** | **0,15** | **0,05** |

| **Prot OPG : Kidney ACE2** | | |  | TaqMan |  |  |  |  |  |
| --- | --- | --- | --- | --- | --- | --- | --- | --- | --- |
|  |  |  |  |  |  |  |  |  |  |
| **Treatment** | **FAM** | **VIC** | **dCt** | **Av cont.** | **dd ct** | **fold incr.** | **average** | **St.Dev** | **SEM** |
| CNT | 24,44 | 9,80 | 14,64 |  | -0,30 | 1,23 |  |  |  |
| CNT | 24,64 | 9,41 | 15,23 |  | 0,29 | 0,82 |  |  |  |
| CNT | 24,59 | 9,42 | 15,17 |  | 0,24 | 0,85 |  |  |  |
| CNT | 24,18 | 9,41 | 14,76 |  | -0,17 | 1,13 |  |  |  |
| CNT | 24,18 | 9,42 | 14,76 |  | -0,18 | 1,13 |  |  |  |
| CNT | 24,42 | 9,38 | 15,04 |  | 0,11 | 0,93 |  |  |  |
| CNT | 24,38 | 9,43 | 14,94 | **14,93** | 0,01 | 0,99 | **1,01** | **0,16** | **0,06** |
| OPG | 24,10 | 9,23 | 14,87 |  | -0,07 | 1,05 |  |  |  |
| OPG | 24,26 | 9,42 | 14,84 |  | -0,09 | 1,07 |  |  |  |
| OPG | 24,35 | 9,48 | 14,87 |  | -0,06 | 1,04 |  |  |  |
| OPG | 24,50 | 9,47 | 15,03 |  | 0,09 | 0,94 |  |  |  |
| OPG | 24,44 | 9,56 | 14,88 |  | -0,05 | 1,04 |  |  |  |
| OPG | 24,59 | 9,46 | 15,14 |  | 0,21 | 0,87 |  |  |  |
| OPG | 24,57 | 9,44 | 15,13 |  | 0,19 | 0,88 | **0,98** | **0,09** | **0,03** |

| **Prot OPG : Kidney AT1R** | |  |  | TaqMan |  |  |  |  |  |
| --- | --- | --- | --- | --- | --- | --- | --- | --- | --- |
|  |  |  |  |  |  |  |  |  |  |
| **Treatment** | **FAM** | **VIC** | **dCt** | **Av cont.** | **dd ct** | **fold incr.** | **average** | **St.Dev** | **SEM** |
| CNT | 29,89 | 9,14 | 20,75 |  | -0,201 | 1,15 |  |  |  |
| CNT | 29,30 | 9,14 | 20,16 |  | -0,791 | 1,73 |  |  |  |
| CNT | 29,32 | 9,14 | 20,18 |  | -0,764 | 1,70 |  |  |  |
| CNT | 30,04 | 9,20 | 20,84 |  | -0,110 | 1,08 |  |  |  |
| CNT | 30,94 | 9,23 | 21,72 |  | 0,772 | 0,59 |  |  |  |
| CNT | 30,39 | 9,29 | 21,10 |  | 0,151 | 0,90 |  |  |  |
| CNT | 31,06 | 9,17 | 21,89 | **20,95** | 0,944 | 0,52 | **1,09** | **0,48** | **0,18** |
| OPG | 30,18 | 9,11 | 21,08 |  | 0,128 | 0,91 |  |  |  |
| OPG | 30,26 | 9,07 | 21,20 |  | 0,250 | 0,84 |  |  |  |
| OPG | 30,57 | 9,08 | 21,48 |  | 0,537 | 0,69 |  |  |  |
| OPG | 29,60 | 9,24 | 20,37 |  | -0,581 | 1,50 |  |  |  |
| OPG | 30,58 | 9,28 | 21,30 |  | 0,350 | 0,78 |  |  |  |
| OPG | 29,75 | 9,69 | 20,06 |  | -0,886 | 1,85 |  |  |  |
| OPG | 29,88 | 9,39 | 20,50 |  | -0,448 | 1,36 |  |  |  |
| OPG | 29,70 | 9,23 | 20,47 |  | -0,480 | 1,39 | **1,17** | **0,42** | **0,15** |

| **Prot OPG : Kidney IL6** | |  |  | Sybr |  |  |  |  |  |
| --- | --- | --- | --- | --- | --- | --- | --- | --- | --- |
|  |  |  |  |  |  |  |  |  |  |
| **Treatment** | **IL6** | **Rps9** | **dCt** | **Av cont.** | **dd ct** | **fold incr.** | **average** | **St.Dev** | **SEM** |
| CNT | 32,25 | 18,15 | 14,09 |  | -0,34 | 1,27 |  |  |  |
| CNT | 32,25 | 18,10 | 14,15 |  | -0,29 | 1,22 |  |  |  |
| CNT | 31,94 | 17,78 | 14,16 |  | -0,28 | 1,21 |  |  |  |
| CNT | 33,29 | 17,95 | 15,34 | **14,43** | 0,90 | 0,53 | **1,06** | **0,35** | **0,18** |
| OPG | 31,04 | 17,74 | 13,30 |  | -1,14 | 2,20 |  |  |  |
| OPG | 31,36 | 17,91 | 13,45 |  | -0,98 | 1,98 |  |  |  |
| OPG | 31,61 | 18,28 | 13,33 |  | -1,10 | 2,15 |  |  |  |
| OPG | 31,55 | 18,07 | 13,48 |  | -0,96 | 1,94 |  |  |  |
| OPG | 32,32 | 18,05 | 14,27 |  | -0,16 | 1,12 | **1,88** | **0,44** | **0,20** |

| **Prot OPG : Kidney MCP1** | | |  | TaqMan |  |  |  |  |  |
| --- | --- | --- | --- | --- | --- | --- | --- | --- | --- |
|  |  |  |  |  |  |  |  |  |  |
| **Treatment** | **FAM** | **VIC** | **dCt** | **Av cont.** | **dd ct** | **fold incr.** | **average** | **St.Dev** | **SEM** |
| CNT | 30,33 | 9,27 | 21,05 |  | 0,00 | 1,00 |  |  |  |
| CNT | 30,49 | 9,24 | 21,25 |  | 0,20 | 0,87 |  |  |  |
| CNT | 30,54 | 9,14 | 21,40 |  | 0,35 | 0,78 |  |  |  |
| CNT | 29,59 | 8,89 | 20,69 |  | -0,35 | 1,28 |  |  |  |
| CNT | 30,34 | 8,99 | 21,35 |  | 0,30 | 0,81 |  |  |  |
| CNT | 29,86 | 9,31 | 20,55 | **21,05** | -0,50 | 1,42 | **1,03** | **0,26** | **0,11** |
| OPG | 30,06 | 9,22 | 20,84 |  | -0,21 | 1,16 |  |  |  |
| OPG | 29,52 | 9,14 | 20,38 |  | -0,67 | 1,59 |  |  |  |
| OPG | 30,02 | 9,03 | 20,99 |  | -0,06 | 1,04 |  |  |  |
| OPG | 28,84 | 9,40 | 19,44 |  | -1,61 | 3,06 |  |  |  |
| OPG | 29,32 | 9,49 | 19,83 |  | -1,22 | 2,33 |  |  |  |
| OPG | 30,22 | 9,82 | 20,40 |  | -0,65 | 1,57 | **1,79** | **0,77** | **0,31** |

| **Prot OPG : Kidney TNFa** | |  |  | Sybr |  |  |  |  |  |
| --- | --- | --- | --- | --- | --- | --- | --- | --- | --- |
|  |  |  |  |  |  |  |  |  |  |
| **Treatment** | **TNFa** | **Rps9** | **dCt** | **Av cont.** | **dd ct** | **fold incr.** | **average** | **St.Dev** | **SEM** |
| CNT | 27,45 | 17,65 | 9,80 |  | -0,39 | 1,31 |  |  |  |
| CNT | 28,17 | 18,15 | 10,02 |  | -0,17 | 1,13 |  |  |  |
| CNT | 28,28 | 18,10 | 10,18 |  | -0,02 | 1,01 |  |  |  |
| CNT | 28,10 | 17,78 | 10,32 |  | 0,13 | 0,92 |  |  |  |
| CNT | 28,60 | 17,95 | 10,64 | **10,19** | 0,45 | 0,73 | **1,02** | **0,22** | **0,10** |
| OPG | 27,97 | 17,85 | 10,12 |  | -0,07 | 1,05 |  |  |  |
| OPG | 27,77 | 17,74 | 10,03 |  | -0,16 | 1,12 |  |  |  |
| OPG | 27,80 | 17,79 | 10,01 |  | -0,18 | 1,13 |  |  |  |
| OPG | 27,65 | 17,91 | 9,74 |  | -0,45 | 1,37 |  |  |  |
| OPG | 27,54 | 18,28 | 9,26 |  | -0,93 | 1,90 |  |  |  |
| OPG | 26,77 | 18,07 | 8,69 |  | -1,50 | 2,82 |  |  |  |
| OPG | 27,42 | 18,05 | 9,38 |  | -0,81 | 1,76 | **1,59** | **0,64** | **0,24** |

| **Prot OPG : Kidney CTGF** | | |  | TaqMan |  |  |  |  |  |
| --- | --- | --- | --- | --- | --- | --- | --- | --- | --- |
|  |  |  |  |  |  |  |  |  |  |
| **Treatment** | **FAM** | **VIC** | **dCt** | **Av cont.** | **dd ct** | **fold incr.** | **average** | **St.Dev** | **SEM** |
| CNT | 22,23 | 9,40 | 12,82 |  | -0,11 | 1,08 |  |  |  |
| CNT | 22,32 | 9,62 | 12,69 |  | -0,24 | 1,18 |  |  |  |
| CNT | 22,24 | 9,13 | 13,11 |  | 0,18 | 0,88 |  |  |  |
| CNT | 21,84 | 9,12 | 12,73 |  | -0,20 | 1,15 |  |  |  |
| CNT | 22,41 | 9,39 | 13,02 |  | 0,09 | 0,94 |  |  |  |
| CNT | 22,29 | 9,37 | 12,92 |  | -0,01 | 1,00 |  |  |  |
| CNT | 22,71 | 9,50 | 13,21 | **12,93** | 0,28 | 0,83 | **1,01** | **0,13** | **0,05** |
| OPG | 22,27 | 9,26 | 13,01 |  | 0,09 | 0,94 |  |  |  |
| OPG | 21,94 | 9,26 | 12,69 |  | -0,24 | 1,18 |  |  |  |
| OPG | 21,65 | 9,37 | 12,29 |  | -0,64 | 1,56 |  |  |  |
| OPG | 22,09 | 9,56 | 12,52 |  | -0,41 | 1,33 |  |  |  |
| OPG | 21,65 | 9,40 | 12,25 |  | -0,68 | 1,60 | **1,32** | **0,27** | **0,12** |

| **Prot OPG : Kidney Fibronectin** | | |  | TaqMan |  |  |  |  |  |
| --- | --- | --- | --- | --- | --- | --- | --- | --- | --- |
|  |  |  |  |  |  |  |  |  |  |
| **Treatment** | **FAM** | **VIC** | **dCt** | **Av cont.** | **dd ct** | **fold incr.** | **average** | **St.Dev** | **SEM** |
| CNT | 27,37 | 9,50 | 17,87 |  | -0,01 | 1,01 |  |  |  |
| CNT | 27,67 | 9,58 | 18,09 |  | 0,21 | 0,86 |  |  |  |
| CNT | 27,22 | 9,54 | 17,69 |  | -0,19 | 1,14 |  |  |  |
| CNT | 26,81 | 9,35 | 17,45 |  | -0,42 | 1,34 |  |  |  |
| CNT | 27,70 | 9,47 | 18,22 |  | 0,35 | 0,79 |  |  |  |
| CNT | 26,91 | 9,40 | 17,52 |  | -0,36 | 1,28 |  |  |  |
| CNT | 27,75 | 9,45 | 18,30 | **17,88** | 0,43 | 0,74 | **1,02** | **0,24** | **0,09** |
| OPG | 27,14 | 9,44 | 17,70 |  | -0,17 | 1,13 |  |  |  |
| OPG | 27,16 | 9,45 | 17,72 |  | -0,16 | 1,12 |  |  |  |
| OPG | 27,19 | 9,45 | 17,75 |  | -0,13 | 1,10 |  |  |  |
| OPG | 26,41 | 9,44 | 16,97 |  | -0,91 | 1,87 |  |  |  |
| OPG | 27,26 | 9,47 | 17,79 |  | -0,09 | 1,06 |  |  |  |
| OPG | 26,49 | 9,72 | 16,78 |  | -1,10 | 2,15 | **1,40** | **0,48** | **0,20** |

| **Prot OPG : Kidney TGFbeta** | | |  | Sybr |  |  |  |  |  |
| --- | --- | --- | --- | --- | --- | --- | --- | --- | --- |
|  |  |  |  |  |  |  |  |  |  |
| **Treatment** | **TGFb** | **Rps9** | **dCt** | **Av cont.** | **dd ct** | **fold incr.** | **average** | **St.Dev** | **SEM** |
| CNT | 21,58 | 17,65 | 3,93 |  | 0,20 | 0,87 |  |  |  |
| CNT | 21,76 | 18,15 | 3,61 |  | -0,12 | 1,09 |  |  |  |
| CNT | 21,75 | 18,10 | 3,65 |  | -0,08 | 1,06 |  |  |  |
| CNT | 21,58 | 17,82 | 3,76 |  | 0,03 | 0,98 |  |  |  |
| CNT | 21,53 | 18,03 | 3,51 |  | -0,22 | 1,17 |  |  |  |
| CNT | 21,37 | 17,78 | 3,58 |  | -0,15 | 1,11 |  |  |  |
| CNT | 22,03 | 17,95 | 4,08 | **3,73** | 0,35 | 0,79 | **1,01** | **0,14** | **0,05** |
| OPG | 21,46 | 17,85 | 3,62 |  | -0,12 | 1,08 |  |  |  |
| OPG | 21,32 | 17,74 | 3,57 |  | -0,16 | 1,11 |  |  |  |
| OPG | 21,34 | 17,79 | 3,55 |  | -0,18 | 1,14 |  |  |  |
| OPG | 21,36 | 17,91 | 3,45 |  | -0,28 | 1,21 |  |  |  |
| OPG | 21,62 | 18,28 | 3,34 |  | -0,39 | 1,31 |  |  |  |
| OPG | 21,39 | 18,07 | 3,32 |  | -0,41 | 1,33 |  |  |  |
| OPG | 21,26 | 18,05 | 3,22 |  | -0,51 | 1,43 | **1,23** | **0,13** | **0,05** |

**3. Glomerular nitrotyrosine staining:**

| **Treatment** | **% stained area** | **average** | **St.Dev** | **SEM** |
| --- | --- | --- | --- | --- |
| CNT | 21,63 |  |  |  |
| CNT | 12,12 |  |  |  |
| CNT | 10,13 |  |  |  |
| CNT | 17,77 |  |  |  |
| CNT | 13,63 |  |  |  |
| CNT | 19,18 |  |  |  |
| CNT | 15,28 |  |  |  |
| CNT | 18,20 |  |  |  |
| CNT | 20,47 | **16,49** | **3,92** | **1,31** |
| OPG | 21,00 |  |  |  |
| OPG | 17,31 |  |  |  |
| OPG | 23,39 |  |  |  |
| OPG | 20,85 |  |  |  |
| OPG | 18,20 |  |  |  |
| OPG | 27,05 |  |  |  |
| OPG | 16,29 |  |  |  |
| OPG | 25,65 |  |  |  |
| OPG | 24,33 | **21,57** | **3,80** | **1,27** |
